# The Prevalence of Rhesus-Negative Blood Group Among Patient With Small Cell Lung Cancer and Analysis of Its Effect on Overall Survival

**DOI:** 10.3389/fonc.2018.00358

**Published:** 2018-09-10

**Authors:** Abhishek Biswas, Yue Jiang, Danmeng Li, Hiren J. Mehta, Frederic Kaye

**Affiliations:** ^1^Division of Pulmonary and Critical Care Medicine, Department of Internal Medicine, University of Florida, Gainesville, FL, United States; ^2^Interdisciplinary Program in Biomedical Sciences, University of Florida, Gainesville, FL, United States; ^3^Department of Health Outcomes & Policy, College of Medicine, Gainesville, FL, United States; ^4^UF Health Medical Oncology, Gainesville, FL, United States

**Keywords:** small cell lung cancer, rhesus blood group, overall survival, lung cancer, racial disparity

## Abstract

A higher incidence of Rhesus group D (RHD)-negative blood group among patients with Small Cell Cancer of the lung (SCLC) had been previously reported but reproducibility was not confirmed, and clinical relevance is undefined. We tested 1,090 (SCLC; Adenocarcinoma: Squamous = 202:536:352) cases of lung cancer over a 3-year period at a single institution and noted a higher frequency RHD negative status among SCLC cases (19/89) compared with non-SCLC (61/480) that could not be explained by differences in ethnic background in the patient population. While we confirmed poor ECOG functional status, advanced stage, elevated alkaline phosphatase, and low albumin levels as independent and significant factors for reduced overall survival (OS), we did not detect any clinical outcome correlations with RHD status in our dataset. Patients with SCLC rarely undergo surgical resection resulting in limited data for blood group analyses. We have now detected a higher rate of RHD-negative status in patients with SCLC compared with all other subtypes of lung cancer. The clinical and biological basis for this observation is undefined and we feel that this may be explained by variations in ethnic background.

## Introduction

Small cell lung cancer (SCLC) is an aggressive neuroendocrine subtype with poor prognosis (1). Since patients with SCLC rarely undergo surgical resection, there have been few studies on the genetic landscape of somatic mutations in primary SCLC tumors as compared to other histologic subtypes. While performing genome sequencing of 19 patients with SCLC, we noticed the occurrence of deletion or misreads within the RHD locus in 6/19 samples. A literature review on this topic indicated that rhesus negative (RHD–ve) blood group was more commonly observed in patients diagnosed with SCLC than would be expected based on its incidence in the general population (2). Studies on non-small cell lung cancer subtypes have shown that a relationship between the expression of Rhesus antigens on blood erythrocytes and overall survival may exist among patients having adenocarcinoma of the lung. However, such a relationship was not reported with non-adenocarcinoma lung cancers types in that report (3). An inconsistent relationship has been reported to exist between Rhesus blood group and outcomes in SCLC. Oguz et al. did not find any difference in RHD status and lung cancer histological subtypes in their analysis of 221 patients (4). Similar results have been reported by Cerny et al. (5). On the other hand, Urun et al. reported an association between RHD–ve blood group and a higher risk of lung cancer (6). However, their analysis did not look at different histological lung-cancer subtypes. In the absence of well-designed studies with large number of patients, it becomes difficult to conclude if the higher observed frequency of RHD–ve blood group status in patients with SCLC is clinically relevant or just an association by chance. We have now studied 569 cases of lung cancer including 89 SCLC to test if there is an association between RHD status and overall survival among these patients.

## Methods and materials

We reviewed records of all lung cancer patients receiving treatment at the University of Florida Cancer center between 2012 and 2015 (*N* = 1,090). The Institutional Review Board at the University of Florida approved the study. Patient demographic information, ABO & Rh blood group, and clinical features were reviewed. Demographic and clinical variables were analyzed for impact on overall survival (Kaplan-Meier method plus log-rank test) with < 0.05 taken as being significant for inclusion in multivariate analysis (Cox regression). Rhesus group determination was tested using known antisera, specifically Anti-D IgG. With the gel test, D antigen typing are performed using microtubes which has the specific antisera Anti-D incorporated within the gel. Agglutination indicates the presence of an antigen-antibody reaction, while lack of agglutination indicates the absence of an antigen-antibody reaction.

## Results

**Figure 1:**
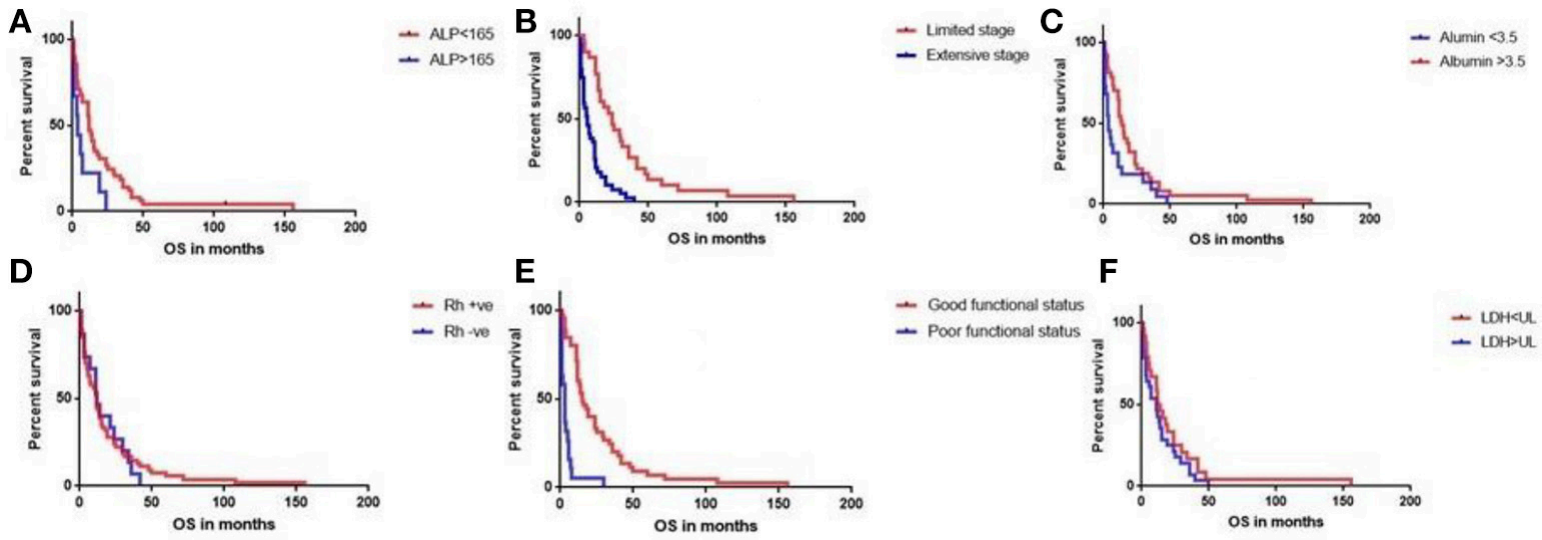
Log-rank (Mantel-Cox) test did not detect any effect of RHD status on overall survival (P0.8) **(D)**. Serum alkaline phosphatase level (ALP) above 165 U/L **(A)**, extensive stage SCLC **(B)**, serum albumin < 3.5 g/dL **(C)**, poor functional status (ECOG 2–4) **(E)** were associated with poor survival. LDH levels above 225 IU/L were not found to be predictive of a poor survival **(F)**.

Out of 1,090 cases of lung cancer, 569 had blood group data available. This included 480/808 patients with Non-small cell cancer and 89/202 cases of SCLC. RHD–ve SCLC patients accounted for 21.35% of all SCLC (19/89). This was significantly higher than those with non-SCLC group (61/480 or 12.70%; *P* = 0.04 by Fishers' exact *t*-test) (Table 1). Table 2 demonstrates the demographic data. Of these, 69 had complete data making them eligible for final statistical analysis and 15/69 (21.7%) had RHD–ve blood group. We then evaluated the data available for these 69 patients and checked for statistically significant variables. We found that Functional status (ECOG), serum Alkaline Phosphatase level above 165 IU/L, stage of the disease (limited vs. extensive), response to treatment (responders' vs. non-responders) and serum albumin levels of 3.5 and above predicted overall survival (OS) whereas RHD–ve blood group did not influence overall survival (Figure 1).

**Table 1:** Tumor histology and RHD status.

**Cancer type**	**Squamous cell cancer**	**Adenocarcinoma**	**SCLC**	**Total**
Total patients	130	243	89	569
RhD –ve	17	29	19	80
% of RhD –ve	13.08%	11.93%	21.35%	14.06%

**Table 2:** Demographic information for all, RHD +ve and RHD –ve patients.

**Characteristic**	**All (*N* = 69)**	**RHD +ve (*N* = 54)**	**RHD –ve (*N* = 15)**	***p*-value**
Mean (Standard Deviation)				
Age at diagnosis,	62.77 (9.09)	62.93 (9.20)	62.20 (8.94)	0.7
years				
Overall Survival	19.24 (25.32)	19.96 (27.76)	16.67 (13.67)	0.5
period, months				
Number (%)				
Race/ethnicity	69 (100)	54 (78.26)	15 (21.74)	0.4
Caucasian	63 (91.30)	50 (92.59)	13 (86.67)	
African American	6 (8.70)	4 (7.41)	2 (13.33)	
Sex (Male)	27 (39.13)	20 (37.04)	7 (46.67)	0.4
Age>65	26 (37.68)	21 (39.62)	5 (33.33)	
ABO Type	69 (100)	54 (78.26)	15 (21.74)	
A	23 (33.33)	15 (27.78)	8 (53.33)	0.1
B	7 (10.14)	7 (12.96)	0 (0.00)	
AB	2 (2.90)	2 (3.70)	0 (0.00)	
O	37 (53.62)	30 (55.56)	7 (46.67)	
ECOG	69 (100)	53 (76.8)	16 (23.1)	
0–1	23 (33.4)	17 (32.00)	6 (37.5)	0.7
2–4	46 (66.6)	36 (68.00)	9 (56.25)	
Chemoradiation (yes)	33 (47.83)	26 (48.15)	7 (46.67)	0.9
Smoking (yes)	66 (98.51)	51 (98.08)	15 (100.00)	0.5
Extensive cancer (yes)	39 (56.52)	31 (57.41)	8 (53.33)	0.7
Brain metastasis (yes)	32 (46.38)	26 (50.00)	6 (40.00)	0.4
Distant recurrence (vs. Overall recurrence)	28 (80.00)	25 (86)	3 (50.00)	0.07

Some studies have previously reported fewer number of subjects of African descent among patients with SCLC (7) however others have not confirmed this ethnic association (8). We tested our patient dataset and did observe a lower frequency of patients of African descent among our SCLC cases (9% of SCLC patients; 18/202) compared to adenocarcinoma (75/536, 14%; *P* = 0.08) and squamous cell lung cancer 49/352 (14%; 0.04).

## Discussion

Our cohort of SCLC patients had a significantly higher percentage of patients with RHD–ve blood group status compared those with other forms of lung cancer and the general Caucasian population as reported before (9, 10). However, no association between Rh blood group and overall survival in patients with SCLC was detected. Race-comparisons between SCLC and NSCLC cancers showed that significantly more Caucasians tended to have SCLC compared to non-hispanic blacks. This is probably in keeping with the trend noted in the US where the incidence gaps between whites and blacks have been increasing with each decade with SCLC incidence in blacks decreasing over the time (7). Blacks have a lower frequency (3–5%) of RHD- blood group (11–14) compared to the general Caucasian population (15%) (2). This relative lack of black patients (group with lower proportion of RHD–ve blood group) in the SCLC cohort could potentially explain the relatively higher incidence of RHD–ve blood group in the SCLC cohort compared to the NSCLC cohort which has a higher representation of blacks. A higher tendency for distant recurrence of the disease was noted among those with RHD+ blood group compared to those who were RHD–. Whether that actually indicates a protective effect against distant recurrence for those who were RHD– is not clear. This would a need larger study to clarify. Blacks have been reported to have better crude survival rates than white patients only with small cell cancer (15). Again, larger studies would be necessary to determine if the difference in RHD blood group status has any impact on this. There are suggestions that chromosomal rearrangements on the short arm of chromosome 1 (location of the Rh gene locus) may have a role to play in the development of oral, head and neck and colorectal cancers (16, 17). Among other chromosomes, clustering of breakpoint in chromosome 1 has been detected in SCLC specimens (18). Although signals exist to indicate an interaction between Rhesus blood group and different cancers including SCLC, the nature of such an interaction is unclear.

## Limitations

A small sample size, using antisera against only D antigen, thereby limiting further segregation of different Rh phenotypes between those who are labeled as RHD- status are all valid limitations of the study. There are different mechanisms accounting for the lack of D antigen expression ranging from deletion of the gene coding the D antigen (common in Caucasians) to a defect at the transcriptional level with a normal gene, as seen in blacks. Hence, the phenotypic expression of Rh status may not be a convincing representation of the underlying genotype and thus may mask a truly significant relationship between Rh status and SCLC. In addition, our study being a retrospective study is not free from a selection bias.

## Conclusion

Rhesus blood group distribution is skewed toward a higher RHD–ve occurrence in patients with SCLC compared to other forms of NSCLC lung cancer but does not appear to have any impact on overall survival. A disproportionately low representation of blacks in the SCLC cohort (who have a significantly lower incidence of RHD-blood group compared to Caucasians) could potentially explain the difference in distribution of the RHD blood groups between SCLC and NSCLC.

## Ethics statement

All procedures performed in studies involving human participants were in accordance with the ethical standards of the institutional and/or national research committee and with the 1964 Helsinki declaration and its later amendments or comparable ethical standards. This was a retrospective study and did not include any actual patients other than clinical data that was already collected as a part of their medical care provided in the past.

## Author contributions

AB, YJ, HM, and FK contributed to study design, collection of data, and writing of manuscript. DL contributed to writing of manuscript.

### Conflict of interest statement

The authors declare that the research was conducted in the absence of any commercial or financial relationships that could be construed as a potential conflict of interest.
